# Supplementary material for: New Therapeutic Targets TIGIT, LAG-3 and TIM-3 in the Treatment of Advanced, Non-Small-Cell Lung Cancer
Source: Int J Mol Sci. 2025 Apr 25;26(9):4096. doi: 10.3390/ijms26094096 (PMC12072094; doi:10.3390/ijms26094096)
Supplement: Supplementary file 1 [file ijms-26-04096-s001.zip › ijms-3549311-supplementary.pdf]

Table S1. Review of selected studies on TIGIT.

**Vibostolimab**	KEYVIBE-002 [1]	Phase II	• NSCLC recurrent or metastatic<br>• 3+ line of treatment<br>• disease progression after platinum doublet chemotherapy and anti–PD-(L)1 inhibitor<br>• without prior docetaxel<br>• vibostolimab/ pembrolizumab + docetaxel vs vibostolimab/ pembrolizumab vs docetaxel + placebo	Study completion date: August 2027
	KEYVIBE-003 [1]	Phase III	• NSCLC recurrent or metastatic<br>• 1 line treatment<br>• PD-L1 TPS ≥1%<br>• Vibostolimab+pembrolizumab vs pembrolizumab	Study completion date: June 2028
	KEYVIBE-006 [1]	Phase III	• Locally advanced and unresectable, nonmetastatic NSCLC<br>• 1 line treatment<br>• vibostolimab/pembrolizumab + platinum doublet chemotherapy + radiotherapy followed by coformulated vibostolimab/pembrolizumab vs platinum doublet chemotherapy + standard radiotherapy followed by durvalumab)	Study completion date: September 2029
	KEYVIBE-007 [1]	Phase III	• VI stage NSCLC<br>• 1 line treatment<br>• vibostolimab/ pembrolizumab + platinum chemotherapy vs pembrolizumab + platinum chemotherapy	Study completion date: September 27, 2027
**Tiragolumab**	CITYSCAPE [2]	Phase II	• recurrent or metastatic NSCLC<br>• chemotherapy-naive,<br>• PD-L1-≥1%<br>• tiragolumab+atezolizumab or placebo plus atezolizumab	PFS 5,4 months in the tiragolumab plus atezolizumab group vs 3,6 months in the placebo plus atezolizumab group
	SKYSCRAPER-02 [3]	Phase III	• I line ES-SCLC.<br>• tiragolumab+atezolizumab+ 4 cycle of chemiotherapy vs placebo plus atezolizumab + 4 cycle of chemiotherapy then maintenance tiragolumab/placebo plus atezolizumab	PFS in the group without of brain metastases 5.4 months tiragolumab v 5.6 months control (P = .3504)<br>OS 13.1 months in both arms
	SKYSCRAPER-01 [4]	Phase III	• I line NSCLC<br>• PD-L1-high, locally advanced or metastatic non-small cell lung cancer (NSCLC)<br>• tiragolumab combined with atezolizumab compared to atezolizumab alone	The study did not reach the primary endpoint of overall survival at the final analysis.
	SKYSCRAPER-06 [5]	Phase II/III	• I line<br>• locally advanced or metastatic NSCLC	The study did not reach the primary endpoint of overall survival

			• Tiragolumab+atezolizumab and chemotherapy as an initial treatment vs pembrolizumab +chemotherapy	and progression-free survival (PFS) at its first interim analysis
**Domvanalimab**	ARC-7 [6,7]	Phase II	• I line<br>• Stage IV, squamous or non-squamous NSCLC with locally assessed high PD-L1 expression (TPS ≥ 50%),<br>• domvanalimab + zimberelimab ± etrumadenant vs monoterapia zimberelimabem.	Median PFS: Zimberlimab: 5.7 mo vs domvanalimab + zimberelimab 12.0 mo vs domvanalimab + zimberelimab +etrumadenant 7,6 mo
	ARC-10 [8]	Phase III	• I-line<br>• locally advanced or metastatic squamous or non-squamous non-small cell lung cancer (NSCLC) with PD-L1 tumor proportion score (TPS) ≥50%<br>• domvanalimab+ zimberelimab vs zimberelimab or chemotherapy	Median PFS (mo) domvanalimab+ zimberelimab arm: 11.5 mo vs zimberelimab arm 6.2 mo vs chemiotherapy arm 9.6 months<br>36% reduction in risk of death in domvanalimab plus zimberelimab arm compared to zimberelimab alone

References

1. Shapira-Frommer, R.; Niu, J.; Perets, R.; Peters, S.; Shouse, G.; Lugowska, I.; Garassino, M.C.; Sands, J.; Keenan, T.; Zhao, B.; et al. The KEYVIBE program: Vibostolimab and pembrolizumab for the treatment of advanced malignancies. *Futur. Oncol.* **2024**, *20*, 1983–1991. https://doi.org/10.1080/14796694.2024.2343272.
2. Cho, B.C.; Abreu, D.R.; Hussein, M.; Cobo, M.; Patel, A.J.; Secen, N.; Lee, K.H.; Massuti, B.; Hiret, S.; Yang, J.C.H.; et al. Tiragolumab plus atezolizumab versus placebo plus atezolizumab as a first-line treatment for PD-L1-selected non-small-cell lung cancer (CITYSCAPE): Primary and follow-up analyses of a randomised, double-blind, phase 2 study. *Lancet Oncol.* **2022**, *23*, 781–792. https://doi.org/10.1016/s1470-2045(22)00226-1.
3. Rudin, C.M.; Liu, S.V.; Soo, R.A.; Lu, S.; Hong, M.H.; Lee, J.-S.; Bryl, M.; Dumoulin, D.W.; Rittmeyer, A.; Chiu, C.-H.; et al. SKYSCRAPER-02: Tiragolumab in Combination with Atezolizumab Plus Chemotherapy in Untreated Extensive-Stage Small-Cell Lung Cancer. *J. Clin. Oncol.* **2024**, *42*, 324–335. https://doi.org/10.1200/jco.23.01363.
4. Roche Reports Update on Phase III SKYSCRAPER-01 Study Results. Available online: https://www.roche.com/media/releases/med-cor-2024-11-26 (accessed on 26 November 2024).
5. Genentech Provides Update of Phase 2/3 Skyscraper-06 Study in Metastatic Nonsquamous Non–Small Cell Lung Cancer. News Release. Genentech. 3 July 2024. Available online: https://www.gene.com/media/press-releases/15029/2024-07-03/genentech-provides-update-on-phase-iiiii (accessed on 11 July 2024).
6. Johnson, M.L. Updates on abstract 397600: ARC-7: Randomized phase 2 study of domvanalimab + zimberelimab ± etrumadenant versus zimberelimab in first-line, metastatic, PD-L1-high non-small cell lung cancer (NSCLC). In Proceedings of the 2023 ASCO Annual Meeting, Chicago, IL, USA, 2–6 June 2023; Abstract 397600.
7. ASCO 2023: Arcus Update on Anti-TIGIT Program & ARC-7 Results. Available online: https://investors.arcusbio.com/investors-and-media/events-and-presentations/corporate-presentation/presentation-details/2023/ASCO-2023-Arcus-Update-on-Anti-TIGIT-Program--ARC-7-Results-2023-h018XgDEp8/default.aspx (accessed on 3 June 2023).
8. Arcus Biosciences. Arcus Biosciences announces that domvanalimab plus zimberelimab improved overall survival in ARC-10, a randomized study in patients with PD-L1-high non-small cell lung cancer. In *News Release*; Arcus Biosciences, Inc.: Hayward, CA, USA, 2024. Available online: https://tinyurl.com/yck58s43 (accessed on 6 November 2024).
